# Zinc and breast cancer risk

**DOI:** 10.1186/1897-4287-10-S4-A6

**Published:** 2012-12-10

**Authors:** Katarzyna Kaczmarek, Anna Jakubowska, Grzegorz Sukiennicki, Magdalena Muszyńska, Katarzyna Jaworska-Bieniek, Katarzyna Durda, Tomasz Huzarski, Pablo Serrano-Fernandez, Tomasz Byrski, Jacek Gronwald, Satish Gupta, Jan Lubiński

**Affiliations:** 1 Pomeranian Medical Univeristy and Read Gene SA, Szczecin, Poland

## 

Zinc is a micronutrient, which is essential for human health, playing role as a cofactor of enzymes such as dehydrogenases, peptidases and component of zinc finger domains. In organism zinc is involved in metabolic pathways, immune processes, maintaining ion balance between other elements. Recently, it has been reported that zinc may play role in chemoprevention, and its level may be associated with cancer risk.

## Aim of the study

The aim of this study was to analyze possible association between serum zinc level and breast cancer risk in BRCA1 mutation carriers and noncarriers.

## Materials and methods

The studies have been performed on 3 independent groups: (1) 119 unselected breast cancer patients matched 1:1 with 119 unaffected controls (carriers of BRCA1 mutation have been excluded), (2) 99 breast cancer cases (serum collected at the moment of diagnosis, before treatment) and 198 matched 1:2 unaffected controls, (3):27 breast cancer cases (serum collected 1-2 years before diagnosis) and 53 controls matched 1:2. All individuals in groups (2) and (3) were carriers of BRCA1 mutation.

Zinc level in serum was measured by inductively coupled plasma mass spectrometry (ICP-MS) using Elan DRC-e ICP-Mass Spectrometer, Perkin Elmer.

## Results

In the group of unselected breast cancer patients, individuals with zinc level 759,64-985,24 μg/L had a lower risk of breast cancer than those with zinc level 107,18–759,64 μg/L. Observation was statistically insignificant (OR=0,712; p=0,34).

In group of BRCA1 carriers, in which serum was collected at the moment of diagnosis, individuals with zinc level 692,48 – 756,01 μg/L had significantly lower risk of breast cancer than those with zinc level 218,89 – 692,48 μg/L (OR=0,446, p=0,02709).

In the group of BRCA1 carriers, in which serum was collected 1-2 years before diagnosis, individuals with zinc level >831,35) had significantly lower risk of breast cancer (OR=0,222; p=0,0063).

## Conclusions

We observed that serum zinc level is associated with breast cancer risk in BRCA1 carriers and noncarriers. Analysis of serum zinc concentration revealed tendency to increased risk of breast cancer for unselected breast cancers and BRCA1 carriers with zinc level <750±50 μg/L. Probably high zinc level reduces cancer risk especially in BRCA1 carriers, but these results should be confirmed in larger cohorts.

